# Alternatives to PFASs: Perspectives on the Science

**DOI:** 10.1289/ehp.1509944

**Published:** 2015-05-01

**Authors:** Linda S. Birnbaum, Philippe Grandjean

**Affiliations:** 1 National Institute of Environmental Health Sciences and National Toxicology Program, National Institutes of Health, Department of Health and Human Services, Research Triangle Park, North Carolina, USA; 2 University of Southern Denmark, Odense, Denmark; 3 Harvard School of Public Health, Boston, Massachusetts, USA

Poly- and perfluoroalkyl acids (PFASs) are ubiquitous in our lives. These chemicals are used as surfactants and as water and oil repellents in a variety of consumer products such as cosmetics, food packaging, furnishings, and clothing. Since their initial marketing more than 60 years ago, extensive research has demonstrated that the long-chain PFASs are highly persistent, bioaccumulative, and toxic (Buck et al. 2011). As a result, they are being phased out in many countries. However, controversy has emerged regarding the safety of the most common alternatives, the short-chain PFASs.

In the Madrid Statement on Poly- and Perfluoroalkyl Substances (PFASs), Blum et al. (2015) question the use of the entire class of PFASs, including short-chain fluorinated alternatives. Authored by 14 experts on the health effects, environmental fate, and policy issues concerning PFASs, the Madrid Statement documents the scientific consensus about the extreme environmental persistence, bioaccumulation, and potential toxicity of the overall class of PFASs (Blum et al. 2015). The statement defines a roadmap for scientists, governments, product manufacturers, purchasing organizations, and consumers to work together to limit the production and use of PFASs globally and to develop safer alternatives. Since it was presented at the 34th International Symposium on Halogenated Persistent Organic Pollutants, held 31 August–5 September 2014 in Madrid, Spain, 206 scientists and professionals from 40 countries have signed the Statement (Blum et al. 2015).

In a response to the Madrid Statement in this issue of *EHP*, the FluoroCouncil, which represents the world’s leading fluorotechnology companies, agrees that it “could support many of these policy recommendations if they were limited to long-chain PFASs” (Bowman 2015). The FluoroCouncil supports the call to action from the scientific and professional community to limit the production and environmental release of long-chain PFASs but states that “the short-chain PFAS substances studied to date are not expected to harm human health or the environment,” as they “are eliminated more rapidly from the body and are less toxic than long-chain substances” (Bowman 2015).

Although there is agreement regarding the shorter human half-lives of short-chain PFASs, the Helsingør Statement on PFASs (Scheringer et al. 2014) and other recent publications (Gomis et al. 2015; Wang et al. 2013, 2015) expressed concerns that fluorinated replacements are similar to the PFASs they replaced in terms of their chemical structure, environmental persistence, and hazardous potential for both the environment and humans. Given the fact that research raised concern about the long-chain PFASs for many years before action was taken and that global contamination and toxicity have been documented in the general population (Grandjean and Clapp 2014), potential risks of the short-chain PFASs should be taken into account when choosing replacements for the longer-chain compounds.

There are numerous similar examples of replacements for other chemical classes, in which banned or phased-out chemicals have been replaced with structurally similar chemicals. For example, polychlorinated biphenyls were replaced with chlorinated paraffins (National Toxicology Program 2014), polybrominated diphenyl ethers were replaced with other halogenated flame retardants (Birnbaum and Staskal 2004), and bisphenol A has been replaced with bisphenol S, at least in some applications (Rochester and Bolden 2015). Such straightforward replacement strategies may be cost effective in the short term. However, manufacturers may yet incur costs if the closely related alternative is later found to be as toxic as its predecessor. In fact, there are now multi-stakeholder efforts to improve the choice of alternatives to chemicals of concern (Birnbaum 2013; National Research Council 2014).

It has been difficult to find substitutes that match the function and performance level of PFASs. The chemical and thermal stability of PFASs as well as their hydrophobic and oleophobic properties provide unique material benefits (Buck et al. 2011). Significant innovation is thus required to find functional nonfluorinated alternatives to PFASs. The U.S. Environmental Protection Agency (EPA) recently recognized such innovation by awarding its 2014 Designing Greener Chemicals Award to a halogen-free firefighting foam (U.S. EPA 2014).

The growing global field of chemical alternatives assessment (CAA) provides tools and strategies for identifying compounds, materials, or product designs to substitute for the use of hazardous chemicals (Lavoie et al. 2010). For example, the California Department of Toxic Substances Control is using CAA in its Safer Consumer Products Program, whose objective is to remove toxic chemicals from products (California Department of Toxic Substances Control 2010). Many CAAs have already been conducted, and many more are in progress (e.g., Substitution in Practice of Prioritized Fluorinated Chemicals to Eliminate Diffuse Sources 2015). Conducting CAAs may prove valuable in clarifying the state of the science among potential alternatives to PFASs and providing guidance for future research and innovation. Nevertheless, finding an optimal alternative substance or technology is not straightforward, and CAAs may not always offer solutions. For instance, suitable nonfluorinated alternatives for certain functions of PFASs, such as stain resistance, appear to be lacking or underdeveloped.

Research is needed to understand the potential for adverse health effects from exposure to the short-chain PFASs, especially regarding low-dose endocrine disruption and immunotoxicity. In parallel, research is needed to find safe alternatives for all current uses of PFASs. The question is, should these chemicals continue to be used in consumer products in the meantime, given their persistence in the environment? And, in the absence of indisputably safe alternatives, are consumers willing to give up certain product functionalities, such as stain resistance, to protect themselves against potential health risks? These conundrums cannot be resolved by science alone but need to be considered in an open discussion informed by the scientific evidence.
